# Baseplate inferior offset affects shoulder range of motion in reverse shoulder arthroplasty in Asian population

**DOI:** 10.1186/s13018-023-04506-w

**Published:** 2024-01-03

**Authors:** Erina Yamada, Naoya Kozono, Akira Nabeshima, Eiji Tashiro, Yasuharu Nakashima

**Affiliations:** Department of Orthopaedic Surgery, Graduate School of Medical Sciences, Kyushu University, 3-1-1 Maidashi, Higashi-Ku, Fukuoka, 812-8582 Japan

**Keywords:** Reverse total shoulder arthroplasty, Simulation, Asian, Impingement, Offset

## Abstract

**Background:**

Impingement is a common complication of reverse shoulder arthroplasty. Placement of the baseplate with a wide impingement-free angle is ideal; however, there are few studies on Asian populations, which have smaller height and physique, and there is a lack of guidance on achieving optimal outcomes. The purpose of the present study was to explore the impingement-free range of motion reverse shoulder arthroplasty and analyze the suitable baseplate position or tilt for the Asian population using simulation software.

**Methods:**

We uploaded computed tomography scan data from 20 Asian patients to three-dimensional (3D) simulation software. The implantation of the reverse shoulder arthroplasty component was performed on the 3D humerus and scapula using software, and range of motion was assessed until impingement occurred.

**Results:**

The range of motion in flexion significantly improved when the baseplate was lowered up to 3 mm inferiorly. Range of motion in abduction and internal and external rotation significantly improved as the baseplate was lowered up to 4 mm. There was no significant difference in range of motion in any motion after changing the inferior tilt, except in internal and external rotation.

**Conclusions:**

The range of motion in abduction, flexion, and internal and external rotations significantly improved with increased inferior offset. These results may prove valuable in determining the optimal baseplate position for RSA, particularly in Asian populations.

## Background

Reverse shoulder arthroplasty (RSA) was first described by Grammont in 1985 as a surgery to improve pain and range of motion (RoM) in patients with cuff-deficient shoulders [1]. Subsequently, the indications for RSA have expanded from rotator cuff tear arthroplasty to proximal humeral fractures, proximal humeral tumors, or revisions, with good results. However, complications have emerged with the increasing number of RSA cases being performed. Impingement of the scapula, humerus, or humeral stem is one example of such complications and is considered a major cause of dislocation, fracture, or loosening of the baseplate [2, 3]. Several studies have reported that lateralization of the humeral stem and distalization of the glenoid component can improve RoM in reverse shoulder arthroplasty [4, 5]; however, there is limited research in this area on Asian populations, and there are few indicators on how to optimize implantation for this demographic. The examination of RoM with a different position or tilt of the baseplate will provide us with useful information for the preoperative planning of RSA.

We aimed to assess the suitable baseplate position or tilt for Asian populations, which have smaller height and physique, and analyze the impact of baseplate parameters on RoM in reverse shoulder arthroplasty, utilizing simulation software. We hypothesized that shifting or tilting the baseplate inferiorly would improve RoM, even in the Asian population.

## Methods

### Participants

The present study was approved by the institutional review board (Approval no.: 22146-00). We enrolled 20 participants (11 males and nine females), with a mean height of 158 cm, mean body weight of 58.5 kg, mean BMI of 23.1, and mean age of 73.1 years. Computed tomography (CT) (Aquilion, Toshiba, Tochigi, Japan) was performed for preoperative examinations. Ten patients underwent RSA for cuff tear arthropathy, and the other 10 underwent arthroscopic repair for massive rotator cuff tears (Table 1).

**Table 1:** Patient demographics

Demographic data	Patients (*n* = 20)
Age at surgery (years)	73.1 ± 6.9
Sex (Male: Female)	11: 9
Height (cm)	158 ± 7.81 cm
Weight (kg)	58.5 ± 14.3 kg
Body mass index (kg/m2)	23.1 ± 4.0 kg/m2
Massive tear/CTA	10/10

Values are presented as mean ± standard deviation

*CTA* Cuff tear arthropathy

### CT scan and implant

An image with the entire humerus in the field of view was required for the software to digitize the image of the humerus; therefore, only patients for whom such images were available were selected for this study, to allow correct analysis of the humeral orientation. Digital imaging and communications in medicine data were uploaded to the three-dimensional (3D) surgical planning software (Zedshoulder; Lexi Co., Ltd., Tokyo, Japan), and a 3D virtual bone model was created using the CT data. Several digitized locations, including the humeral head, lesser/greater tuberosity, and medial/lateral epicondyles, were marked on the axial CT images to ensure accurate component implantation. CT evaluation and implantation were performed by a shoulder fellowship-trained orthopedic surgeon. Virtual implantation was performed with an onlay design component (Equinoxe; Exactech, Inc.; Gainesville, FL, USA), and a Primary Humeral Stem with 145° of neck shaft angle (component angle 132.5°, liner angle 12.5°) was chosen for this study. The humeral component was implanted with 20° of retroversion, and the stem size was individualized for each patient. The glenoid implants were standardized with a 38 mm eccentric glenosphere and a normal baseplate without augmentation.

### RoM simulation

**Fig. 1:**
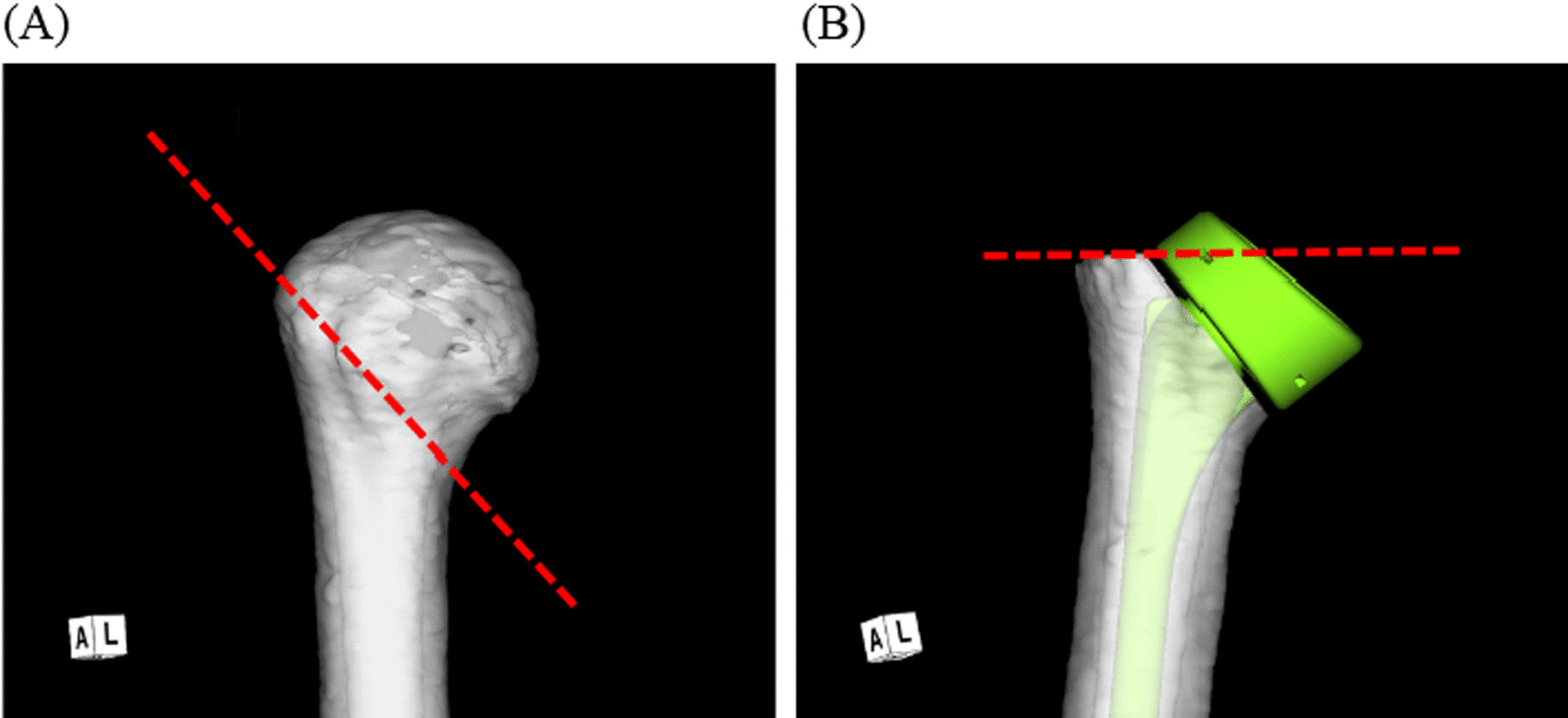
Bone osteotomy. The osteotomy line for the stem is depicted in red. **A** Osteotomy line at inferior margin of the lesser tuberosity. Osteotomy is performed at 132.5° to the humeral bone axis. **B** Osteotomy line of greater tuberosity at the level of the line above the adaptor tray of the stem

**Fig. 2:**
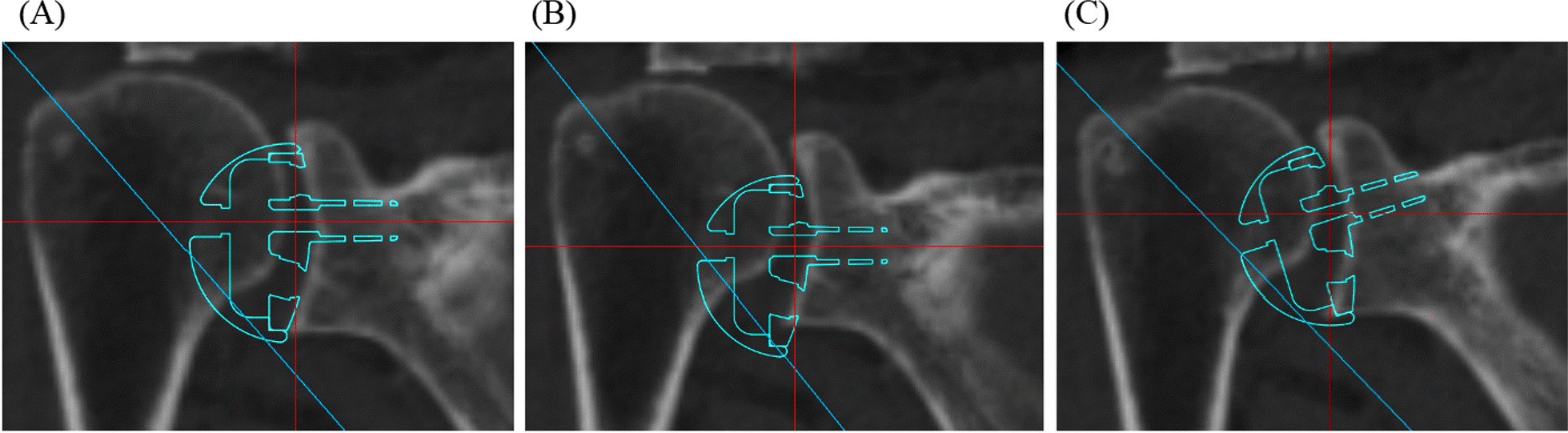
Installation of the baseplate component in the simulation.** A** The bottom of the baseplate and lower surface of the scapula were aligned and determined to have an inferior offset of 0 mm. **B** The baseplate was shifted downward in 1 mm increments from 0 to 5 mm. This figure shows the positioning at 5 mm. **C** The baseplate was tilted inferiorly from 0° to 15° in increments of 5°. This figure shows the title at 15°

**Fig. 3:**
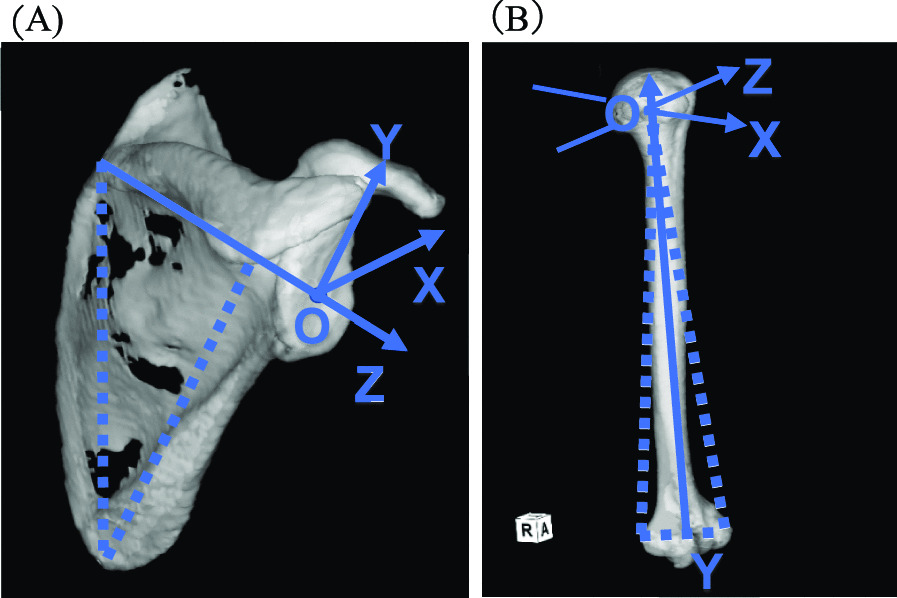
**Axis at the scapula and humerus**. **A** Axis defined by Zedshoulder © at the scapula. O: Midpoint at the anteroposterior direction of the glenoid cavity, X: Perpendicular line to the Z-axis and Y-axis, Y: Parallel line to a perpendicular line drawn from the angulus inferior to the Z-axis, and Z: Line connecting the center point of glenoid and trigonum spinae scapulae. **B** Axis defined by International Society of Biomechanics at the humerus. O: The origin coincident with the glenohumeral rotation center (GH). Y: The line connecting GH and the midpoint of the lateral epicondyle and medial epicondyle. X: A line perpendicular to the plane formed by the lateral epicondyle, medial epicondyle, and GH. Z: A line perpendicular to the Y and X axis

The osteotomy line for the stem was aligned near the inferior margin of the lesser tuberosity, and the greater tuberosity was osteotomized at the level of the line above the adaptor tray of the stem (Fig. 1). The baseplate of the glenoid component was placed on the scapula; the 0 mm position was defined as the position where the lower edge of the baseplate matched the lower edge of the scapula, excluding the osteophytes. Subsequently, the baseplate was shifted downward in 1 mm increments starting from 0 mm till it reached 5 mm (0, 1, 2, 3, 4, and 5 mm). The baseplate inferior tilt angle (0°, 5°, 10°, or 15°) was calculated from the line connecting the trigonum spinae scapulae and the midpoint of the upper and lower maximum diameter of the baseplate and the line perpendicular to the baseplate surface at the midpoint of the upper and lower maximum diameter of the baseplate (Fig. 2). The humeral coordinate system was defined by the International Society of Biomechanics (ISB) [6], and the scapular coordinate system was defined using a software program (Zedshoulder; Lexi Co., Ltd., Tokyo, Japan) (Fig. 3). The humerus moves according to the scapular plane. The X, Y, and Z axes of the humerus and scapula were aligned on the same plane. Flexion/extension was defined as movement around the Z axis, adduction/abduction as movement around the X axis, and internal/external rotation as movement around the Y axis. The RoM for flexion and internal and external rotation was performed with 20° abduction, similar to the native shoulder movement. The software defined impingement as the point where the scapula bone impinged the socket of the humeral prosthesis and the point where the subacromion or coracoid impinged the greater tuberosity. Impingement-free abduction, flexion, and internal and external rotation data were recorded for all the patients.

### Statistical analysis

The RoM was analyzed individually for flexion, abduction, and internal and external rotation by applying inferior offsets of 0, 1, 2, 3, 4, and 5 mm. Additionally, the RoM was examined separately for flexion, abduction, and internal and external rotations at inferior tilt angles of 0°, 5°, 10°, and 15°. The results from all 20 patients were recorded and investigated for statistically significant differences in RoM at each baseplate position. JMP ver.11 (SAS Institute, Cary, NC, USA) was used for all statistical analyses. All data were analyzed using the Wilcoxon signed-rank test for dependent data. *P* < 0.05 was considered statistically significant.

## Results

### Inferior offset

**Fig. 4:**
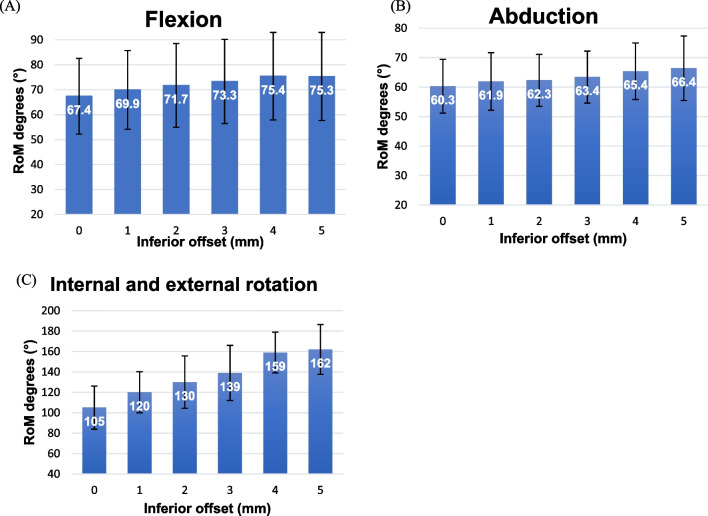
Mean range of motion (RoM) at the inferior offset. Mean RoM in **A** flexion, **B** abduction, and **C** internal and external rotation at each inferior offset (0, 1, 2, 3, 4, and 5 mm). The error bar represents mean ± SD. SD: standard deviation

The mean flexion increased as the inferior offset was raised from 0 to 4 mm; however, between 4 and 5 mm, flexion decreased (Fig. 4A). Shifting the baseplate inferiorly from 0 to 4 mm resulted in an improvement in flexion from 67.4 to 75.4°. We examined flexion by incrementally changing the inferior offset in 1 mm increments from 0 to 5 mm to investigate potential statistically significant differences. Flexion showed a statistically significant increase when the inferior offset was raised from 0 to 1 mm (*P* < 0.001), 1–2 mm (*P* = 0.03), and 2–3 mm (*P* < 0.001) (Table 2). However, there was no significant difference when it was raised from 3 to 4 mm.

**Table 2:** Statistically significant difference between the mean RoM of each baseplate position

Flexion				
Difference (95% CI) *P* value				
0 mm versus 1 mm	0 mm versus 2 mm	0 mm versus 3 mm	0 mm versus 4 mm	0 mm versus 5 mm
2.5 (1.3–3.7) *P* = 0.0004*	4.3 (1.8–6.7) *P* = 0.0017*	5.9 (3.1–8.6) *P* = 0.0003*	8.0 (4.4–11) *P* > 0.0002*	7.9 (3.3–12) *P* = 0.0012*
	1 mm versus 2 mm	1 mm versus 3 mm	1 mm versus 4 mm	1 mm versus 5 mm
	1.7 (0.1–3.3) *P* = 0.0343*	3.3 (1.3–5.3) *P* = 0.0024*	5.4 (2.5–8.3) *P* > 0.0009*	5.3 (1.3–9.3) *P* = 0.0011*
		2 mm versus 3 mm	2 mm versus 4 mm	2 mm versus 5 mm
		1.6 (0.8–2.3) *P* < 0.0001*	3.7 (1.1–6.2) *P* > 0.007*	3.6 (-0.1–7.3) *P* = 0.056
			3 mm versus 4 mm	3 mm versus 5 mm
			2.1 (-0.099–4.2)*P* > 0.060	2.0 (-1.2–5.2) *P* = 0.21
				4 mm versus 5 mm
				0.1 (-1.8–1.6) *P* > 0.90

Abduction				
Difference (95% CI) *P* Value				
0 mm versus 1 mm	0 mm versus 2 mm	0 mm versus 3 mm	0 mm versus 4 mm	0 mm versus 5 mm
1.6 (0.2–2.9) *P* = 0.0018*	2.0 (1.4–2.5) *P* < 0.0001*	3.1 (2.3–3.9) *P* < 0.0001*	5.1 (3.7–6.4) *P* < 0.0001*	6.1 (3.9–8.2) *P* < 0.0001*
	1 mm versus 2 mm	1 mm versus 3 mm	1 mm versus 4 mm	1 mm versus 5 mm
	0.4 (− 0.9 to 1.7) *P* = 0.53	1.5 (0.1–2.9) *P* = 0.034*	3.5 (1.7–5.2) *P* > 0.0006*	4.5 (2.1–6.8) *P* = 0.0007*
		2 mm versus 3 mm	2 mm versus 4 mm	2 mm versus 5 mm
		1.1 (0.7–1.5) *P* < 0.0001*	3.1 (1.9–4.2) *P* < 0.0001*	4.1 (3.1–6.0) *P* = 0.0004*
			3 mm versus 4 mm	3 mm versus 5 mm
			1.9 (0.98–2.9) *P* > 0.0005*	2.9 (1.1–4.7) *P* = 0.002*
				4 mm versus 5 mm
				1.0 (− 0.12 to 2.1) *P* > 0.078

Internal and external rotation				
Difference (95% CI) *P* value				
0 mm versus 1 mm	0 mm versus 2 mm	0 mm versus 3 mm	0 mm versus 4 mm	0 mm versus 5 mm
15 (10–19) *P* < 0.0001*	24 (18–31) *P* < 0.0001*	34 (26–42) *P* < 0.0001*	54 (44–64) *P* < 0.0001*	56 (46–67) *P* < 0.0001*
	1 mm versus 2 mm	1 mm versus 3 mm	1 mm versus 4 mm	1 mm versus 5 mm
	9.9 (5.5–14) *P* = 0.0001*	19 (13–25) *P* < 0.0001*	39 (30–47) *P* < 0.0001*	41 (33–49) *P* < 0.0001*
		2 mm versus 3 mm	2 mm versus 4 mm	2 mm versus 5 mm
		9.7 (4.6–14) *P* = 0.0007*	29 (18–40) *P* < 0.0001*	31 (22–41) *P* < 0.0001*
			3 mm versus 4 mm	3 mm versus 5 mm
			19 (9.9–29) *P* > 0.0005*	22 (14–29) *P* < 0.0001*
				4 mm versus 5 mm
				2.2 (− 7.8 to 6.3) *P* > 0.27

The table indicates the difference between the mean RoM of each baseplate position.

*mean statistically significant difference.

The mean abduction increased as the inferior offset was raised from 0 to 5 mm (Fig. 4B). Shifting the baseplate inferiorly from 0 to 5 mm resulted in an improvement in abduction from 60.3 to 66.4°. Statistically significant differences in abduction were observed when the inferior offset was increased from 0 to 1 mm (*P* = 0.001), 2–3 mm (*P* < 0.001), and 3–4 mm (*P* < 0.001) (Table 2). However, there was no significant differences when it was increased from 4 to 5 mm.

The mean RoM in internal and external rotations increased as the inferior offset was raised from 0 to 5 mm (Fig. 4C). Shifting the baseplate inferiorly from 0 to 5 mm resulted in an improvement in RoM from 105.2° to 150.5°. Statistically significant differences in RoM were observed when the inferior offset increased from 0 to 1 mm (*P* < 0.001), 1–2 mm (*P* < 0.001), 2–3 mm (*P* < 0.001), and 3–4 mm (*P* < 0.001) (Table 2). There was no significant difference when it was increased from 3 to 4 mm.

### Inferior tilt

**Fig. 5:**
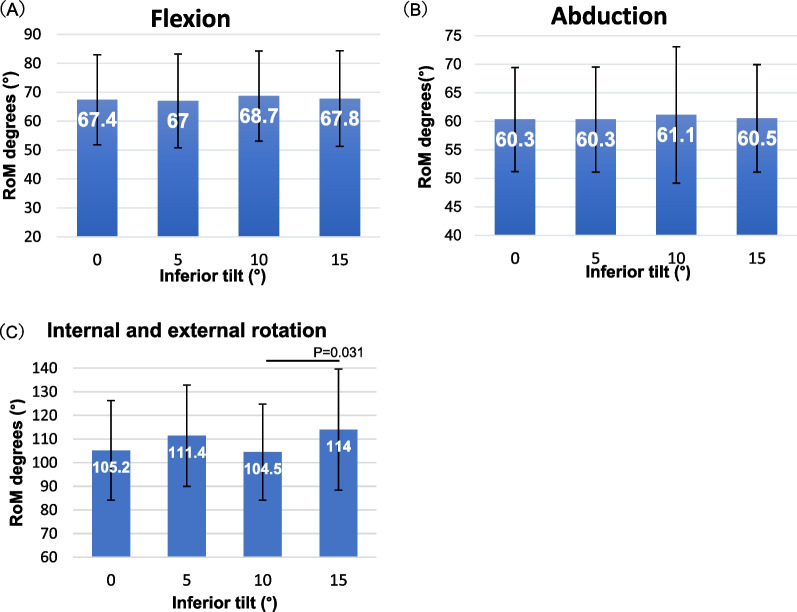
Mean range of motion (RoM) at the inferior tilt. Mean RoM in **A** flexion, **B** abduction, and **C** internal and external rotation at each inferior tilt (0°, 5°, 10°, and 15°). The error bar represents mean ± SD. SD: standard deviation

No statistically significant differences in the RoM were observed during flexion or abduction at any inferior tilt angle. The RoM was significantly larger at 15° than at 10°, during internal and external rotation (Fig. 5).

## Discussion

The present study showed that shoulder flexion statistically significantly increased when the baseplate shifted inferiorly from 0 to 3 mm. Therefore, according to our findings, the optimal inferior offset of the baseplate for flexion could be 3 mm. Similarly, shoulder RoM statistically significantly increased when the baseplate shifted inferiorly in 1 mm increments from 2 up to 4 mm in abduction and from 0 up to 4 mm in internal and external rotation. Therefore, the optimal inferior offset of the baseplate for RoM in abduction and internal and external rotation could be 4 mm.

The present study showed that shoulder abduction tended to improve upon shifting the baseplate inferiorly, but there was no significant difference between 4 and 5 mm. We hypothesized that the greater the inferior offset, the greater the abduction, but our findings did not support this hypothesis. Using computer simulations, Lädermann et al. [7] reported that the acromiohumeral interval (AHI) is strongly correlated with abduction. In the present study, impingement of the greater tuberosity with the subacromion or coracoid process was observed during abduction. The AHI gradually increased as the baseplate was shifted inferiorly, leading to the resolution of the impingement between the subacromion and greater tuberosity, and a smooth passage of the greater tuberosity beneath the acromion. Subsequently, the greater tuberosity passed under the acromion, and then the socket of the prosthesis collided with the upper glenoid due to a further downward shift of the baseplate. Previous studies have also suggested the possibility of similar impingement occurring [8–10]. This impingement persisted in approximately the same position despite shifting further downward and was not eliminated. This may explain why abduction improved with statistical significance in the present study upon shifting of the baseplate inferiorly from 2 to 4 mm but without statistical significance thereafter. Nyffeler et al. [11, 12] also reported abduction; however, in our study, the abduction was generally poorer than that reported in their study. This difference could be attributed to variations in implants, but it may also stem from disparities in populations. Asian populations, especially females, tend to be smaller in height and physique, potentially resulting in a relatively smaller AHI. In the present study, we observed a significant correlation between AHI and the occurrence of greater tuberosity and subacromial impingement. It is hypothesized that Asian populations with a smaller AHI may face challenges in abduction due to this anatomical characteristic.

In flexion, impingement led to a statistically significant improvement in flexion when the baseplate was shifted inferiorly from 0 to 3 mm. The observed flexion was superior to that reported by previous studies [11, 12]. This discrepancy may be attributed to differences in the axes utilized. While both studies moved the humerus in the scapular plane, the axis of the humerus was determined in the present study by ISB [6]. This may have influenced the results in flexion.

The shoulder RoM in internal and external rotations improved with an inferior shifting of the baseplate up to 4 mm of the inferior offset. Bauer et al. [13] reported that the lateralization and inferior overhang of the glenosphere increased the distance between the glenosphere and scapula and improved RoM in internal and external rotation, based on CT scan images of 22 patients. Similarly, the distance between the humerus and scapula increased with an inferior shifting of the baseplate in the present study, suggesting that the increased distance may be the underlying reason for the improved impingement-free internal and external rotation RoM during inferior shifting. Simovitch et al. [14] studied the placement and tilt of the glenosphere in 77 patients to investigate the risk factors for notching and suggested that the inferior offset of the baseplate may prevent impingement of the scapula and lead to improved clinical outcomes, which supports the results of present study. In the present study, the angles of internal and external rotation were found to be larger than those reported in the previous studies. Nyffeler et al. [11, 12] observed an increase in rotation amplitude with higher glenohumeral abduction. It is important to note that, due to the specifications of the Zedshoulder software used in our study, internal and external rotation were calculated with a 20° humeral abduction, potentially contributing to a larger RoM compared to that in other studies.

In the present study, the RoM tended to improve with an inferior shift of the baseplate; therefore, better results than those obtained could have been achieved with a further inferior shift of the baseplate. However, an inferior offset > 5 mm was virtually impossible due to the small scapular fossa in Asians. In most cases, particularly in females, an inferior offset > 5 mm resulted in perforation of the screw or peg from the scapula. This result may be improved by using the recently developed small-type baseplate.

In the present study, an eccentric glenosphere was utilized, and the findings suggest potential benefits in achieving better RoM by using a glenosphere with even greater eccentricity or by increasing the radius of the glenosphere. Collotte et al. [15] proposed that an eccentric glenosphere is associated with less notching compared to a concentric glenosphere. De Biase et al. [16] reported that patients with an eccentric glenosphere demonstrated significantly better flexion and abduction than those with a concentric glenosphere. Berhouet et al. [17] found that a large diameter glenosphere helps prevent scapular notching, while Chou et al. [18] suggested that a larger diameter glenosphere enhances adduction and abduction. Considering the importance of achieving solid fixation, it is ideal for baseplate to cover as much of the glenoid area as possible. Increasing the eccentricity or radius of the glenosphere, rather than solely shifting the baseplate inferiorly, may be advantageous for enhancing stability between the baseplate and the scapula bone. Inferior shifting of the baseplate shifts the center of rotation distally and increases the total humeral length; it has been suggested that increased AHI and humeral length after RSA negatively affect RoM [19]. Another previous study suggested that postoperative humeral lengthening does not affect postoperative outcomes [20]. Excessive extension of the humerus can lead to overextension of the deltoid muscle, leading to complications, such as acromial fractures [21]; therefore, caution must be exercised.

Regarding the inferior tilting of the baseplate in RSA, using the simulation software, Patel et al. [22] reported that the RoM in internal and external rotations was improved with a glenosphere tilt of 0° rather than − 10°, based on CT scan data for 20 shoulders. They suggested that it is a result of the increased medialization necessary to seat an inferiorly tilted implant. Kempton et al. [23] assessed 71 postoperative shoulders on radiographs to determine whether glenosphere tilt was related to scapular notching with neutral tilt and -10° and -15° tilts and found no significant differences among the three tilts. They concluded that an inferior tilt of the glenosphere did not reduce scapular notching. The present study findings also suggest that the baseplate inferior tilt angle may not improve the RoM even in an Asian population.

In the present study, the baseplate was tilted by shaving the lower part of the glenoid without using an augmentation stem or bone graft. Therefore, the greater the inferior tilt, the more the center of rotation was medialized. Using computer simulations of 20 cases, Werner et al. [24] showed that the lateralization of the center of rotation improved the RoM in abduction, flexion, and extension. Using computer simulations with CT data from 12 cases, Lädermann et al. [25] assessed the use of various components of the humerus and scapula and concluded that the lateralization of the center of rotation improved RoM. Therefore, lateralizing the center of rotation is beneficial for RoM. However, in the present study, the center of rotation was medialized, and this may be one reason why RoM did not show a statistically significant improvement.

Initially, it was hypothesized that both inferior tilt and superior tilt may impact RoM in this simulation study; however, recent studies have suggested that the superior tilt has many biomechanical disadvantages, such as increased shear forces and reduced compression forces, which may cause loosening [26–30]. Therefore, only the inferior tilt was evaluated.

In the present study, an inferior tilt was achieved without using augmentation or bone grafts; therefore, placing pegs or screws was difficult due to scapular perforation when the tilt exceeded 15°. Therefore, experiments were performed at tilt angles of 0°, 5°, 10°, and 15°.

### Limitations

In the present study, the degree of shoulder RoM at the point where impingement occurred was recorded. We grouped all cases of impingement together, whether the impingement occurred at the greater tuberosity and subacromion, the greater tuberosity and the coracoid process, or the prosthesis and the glenoid. A more detailed analysis would have been possible if there were more cases. Further studies with larger study samples are needed to validate our results. Additionally, the findings were derived from a simulation software; therefore, the tension of muscles, ligaments, and other soft tissues were ignored. Only the osseous limits of RoM were analyzed and soft tissues were ignored, particularly in internal and external rotations; therefore, some degrees of RoM may be impossible in real patients. Another limitation is that only one type of implant was investigated in the present study; therefore, the results may have differed if another design implant was used. Finally, the standard baseplate was considerably large for the scapula in Asians, particularly for females, so there were some restrictions on implantation. Smaller baseplates may be more suitable for future studies. Further findings may be obtained by using other implant designs and by using a small standard baseplate, especially for Asian females.

## Conclusions

In the present study, the abduction, flexion, and RoM in internal and external rotation showed significant improvement when the baseplate inferior offset was increased in the simulation software for RSA. These results may assist in determining the optimal baseplate position for RSA, particularly in Asian populations.

## Data Availability

The datasets used and/or analyzed during the current study are available from the corresponding author on reasonable request.

## Abbreviations

3D: Three-dimensional
ISB: International Society of Biomechanics
AHI: Acromiohumeral interval
CT: Computed tomography
RoM: Range of motion
RSA: Reverse shoulder arthroplasty
